# Replication interference between human papillomavirus types 16 and 18 mediated by heterologous E1 helicases

**DOI:** 10.1186/1743-422X-11-11

**Published:** 2014-01-24

**Authors:** Seiichiro Mori, Rika Kusumoto-Matsuo, Yoshiyuki Ishii, Takamasa Takeuchi, Iwao Kukimoto

**Affiliations:** 1 Pathogen Genomics Center, National Institute of Infectious Diseases, 4-7-1 Gakuen, Musashi-murayama, Tokyo 208-0011, Japan

**Keywords:** Human papillomavirus, Co-infection, Replication, Interference, E1 helicase

## Abstract

**Background:**

Co-infection of multiple genotypes of human papillomavirus (HPV) is commonly observed among women with abnormal cervical cytology, but how different HPVs interact with each other in the same cell is not clearly understood. A previous study using cultured keratinocytes revealed that genome replication of one HPV type is inhibited by co-existence of the genome of another HPV type, suggesting that replication interference occurs between different HPV types when co-infected; however, molecular mechanisms underlying inter-type replication interference have not been fully explored.

**Methods:**

Replication interference between two most prevalent HPV types, HPV16 and HPV18, was examined in HPV-negative C33A cervical carcinoma cells co-transfected with genomes of HPV16 and HPV18 together with expression plasmids for E1/E2 of both types. Levels of HPV16/18 genome replication were measured by quantitative real-time PCR. Physical interaction between HPV16/18 E1s was assessed by co-immunoprecipitation assays in the cell lysates.

**Results:**

The replication of HPV16 and HPV18 genomes was suppressed by co-expression of E1/E2 of heterologous types. The interference was mediated by the heterologous E1, but not E2. The oligomerization domain of HPV16 E1 was essential for HPV18 replication inhibition, whereas the helicase domain was dispensable. HPV16 E1 co-precipitated with HPV18 E1 in the cell lysates, and an HPV16 E1 mutant Y379A, which bound to HPV18 E1 less efficiently, failed to inhibit HPV18 replication.

**Conclusions:**

Co-infection of a single cell with both HPV16 and HPV18 results in replication interference between them, and physical interaction between the heterologous E1s is responsible for the interference. Heterooligomers composed of HPV16/18 E1s may lack the ability to support HPV genome replication.

## Background

Human papillomavirus (HPV) has a circular double-stranded DNA genome of approximately 8 kilo base-pairs packaged in a capsid composed of two proteins L1 and L2 [1]. HPVs are classified into at least 170 types based on the homology of nucleotide sequences of the L1 gene [2], among which HPV16 and HPV18 are responsible for approximately 70% of cervical cancer cases worldwide.

The life cycle of HPV is tightly linked to the differentiation of host epithelial cells. HPV infects basal cells of stratified epithelia through small lesions, in which the viral genome is maintained as episomes without expressing capsid genes and is passed on to daughter cells. When the host cells initiate epithelial differentiation, the HPV genome starts to replicate, and infectious virions are produced and released from terminally differentiated cornified cells [3].

Productive replication of the HPV genome in differentiated cells requires a viral DNA helicase E1, a viral replication/transcription factor E2, and a replication origin DNA, containing specific binding sites for E1 (E1BS) and E2 (E2BS) [4]. To initiate viral genome replication, E2 binds to the E2BS at the origin and recruits E1, leading to the formation of an E1/E2 complex at the replication origin [4]. Then, E1 changes its conformation by binding to ATP, which causes a release of E2 from the origin. After dissociation of E2, E1 assembles into double trimers to unwind double-stranded DNA. The E1 double trimers serve as precursors for subsequent formation of double hexamers that finally function as a processive DNA helicase at replication forks [5].

E1 consists of four domains: an N-terminal domain (ND), a DNA-binding domain (DBD), an oligomerization domain (OD), and a helicase domain (HD) [4]. The ND contains nuclear localization and export signals, and has a regulatory role in viral genome replication, by controlling subcellular localization of E1 [6]. The DBD is required for E1 binding to the E1BS at the replication origin [7,8]. The OD is responsible for E1-oligomerization [9,10]. The HD has nonspecific DNA-binding and ATPase activities, and mediates interaction with E2 [5,7].

Co-infection with multiple HPV types in a single cervical specimen has been reported in 30–45% of HPV-positive women [11-15]. Furthermore, several studies indicate recombination between different HPV types [16-19], which strongly suggests that a single cell can be co-infected with different HPV types in vivo.

A previous study reported that HPV45 genomes are not maintained in cultured keratinocytes co-transfected with genomes of other HPV types, such as HPV18, HPV31, and HPV39 [20]. This study also demonstrated that while HPV31 and HPV39 genomes are stably maintained within the same cell, copy numbers of genomes of both types are extremely low compared to those in cells containing each genotype individually. These observations suggest an interference of genome replication between different HPV types. However, mechanisms underlying the replication interference have not been fully explored.

In this study, to examine simultaneous replication of HPV16 and HPV18 genomes in the same cell, we used a cell-based transient replication assay that has been used to study E1/E2-dependent HPV replication [21-26]. The HPV-negative C33A cervical cancer cell line was co-transfected with HPV16 and HPV18 genomes together with E1/E2 expression plasmids for both types, and levels of genome replication were measured by quantitative real-time PCR. We report replication interference between HPV16 and HPV18, and propose a molecular mechanism of the interference.

## Results

### Expression of FLAG-tagged E1/E2s of HPV16 and HPV18 in C33A cells

**Figure 1:**
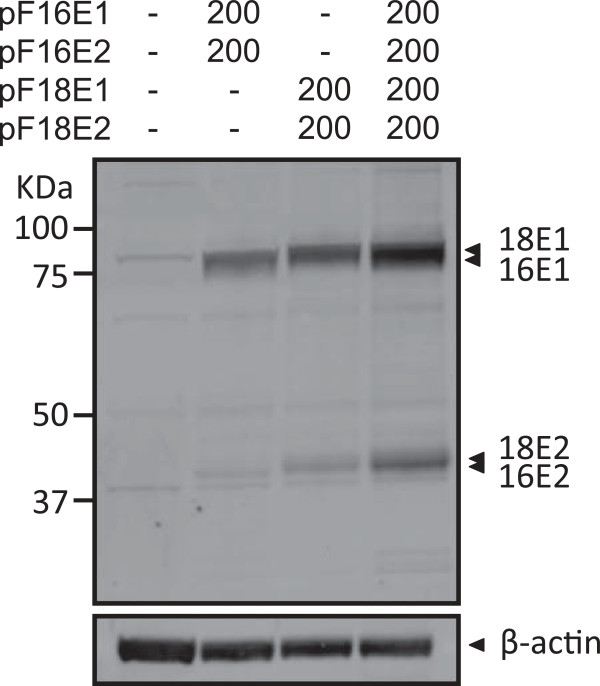
**Expression of FLAG-tagged E1/E2s of HPV16/18 in C33A cells.** C33A cells were transfected with the indicated amounts (ng) of the expression plasmids for E1/E2s of HPV16/18. Two days after transfection, FLAG-tagged E1/E2s in the cell lysates were detected by Western blotting with anti-FLAG antibody. β-actin was detected as loading control.

We constructed expression plasmids for FLAG-tagged HPV16 E1 (F16E1), HPV16 E2 (F16E2), HPV18 E1 (F18E1), and HPV18 E2 (F18E2), and named them: pF16E1, pF16E2, pF18E1, and pF18E2, respectively. Figure 1 shows the expression of the FLAG-tagged E1/E2s in HPV-negative C33A cells transiently transfected with different mixtures of pF16E1 and pF16E2, and/or pF18E1 and pF18E2. The E1/E2 proteins of both types were detected by Western blotting with anti-FLAG antibody. The expression levels of E1/E2 of HPV18 were higher than those of HPV16. Although the expression level of HPV16 E2 was extremely low, the level was sufficient to support HPV16 replication (see below).

### Replication of HPV16 or HPV18 genomes supported by homologous or heterologous E1/E2s

**Figure 2:**
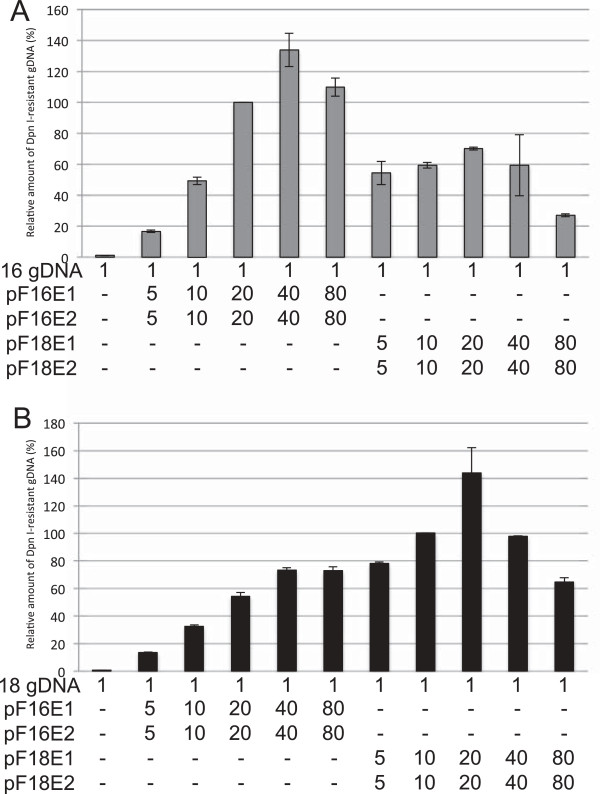
**Replication of HPV16 or HPV18 genomes supported by homologous or heterologous E1/E2s.** C33A cells were transfected with 1 ng of the HPV16 **(A)** or HPV18 **(B)** gDNAs together with the indicated amounts (ng) of the expression plasmids for E1/E2s. Three days after transfection, low molecular weight DNA was isolated by the Hirt procedure and digested with *Dpn*I. The *Dpn*I-resistant HPV gDNA was quantified by real-time PCR and normalized to the luciferase gene. The level of the replication was presented as the relative amount of the *Dpn*I-resistant DNA compared to that obtained by the replication when cells were transfected 20 ng of each pF16E1 and pF16E2 **(A)** or 10 ng of each pF18E1 and pF18E2 **(B)**. Each bar represents the average of two independent experiments with the standard error of mean.

E1/E2s of HPV16 and HPV18 supported genome replication of both the homologous and heterologous types (Figure 2). C33A cells were transfected with circularized full-genome DNA (gDNA) of HPV16 (Figure 2A) or HPV18 (Figure 2B) together with increasing amounts of pF16E1 and pF16E2, or pF18E1 and pF18E2. Three days after transfection, low molecular weight DNA was isolated using the Hirt procedure, digested with *Dpn*I, and *Dpn*I-resistant HPV gDNA quantified by real-time PCR as previously reported [22,25,26]. The maximum levels of replication for HPV16 and HPV18 genomes were observed when cells were transfected with 40 ng of each pF16E1 and pF16E2 and 20 ng of each pF18E1 and pF18E2, respectively. Compared to the replication levels for HPV16 or HPV18 supported by the homologous E1/E2, replication supported by the heterologous E1/E2 was relatively inefficient.

Transfection with excess amounts of the E1/E2 expression plasmids (>80 ng of each pF16E1 and pF16E2, and >40 ng of each pF18E1 and pF18E2) decreased the replication levels for both types (Figure 2) and increased the number of detached cells (data not shown). Since E1 is known to activate a cellular DNA damage response, which results in delayed S phase [27,28], overexpression of E1 may be toxic for C33A cells and reduce the replication efficiency. Therefore, in later experiments, we transfected cells with suboptimal amounts of the expression plasmids (<20 ng of each pF16E1 and pF16E2, and <10 ng of each pF18E1 and pF18E2).

### Simultaneous replication of HPV16 and HPV18 genomes in the presence of E1/E2s of both types

**Figure 3:**
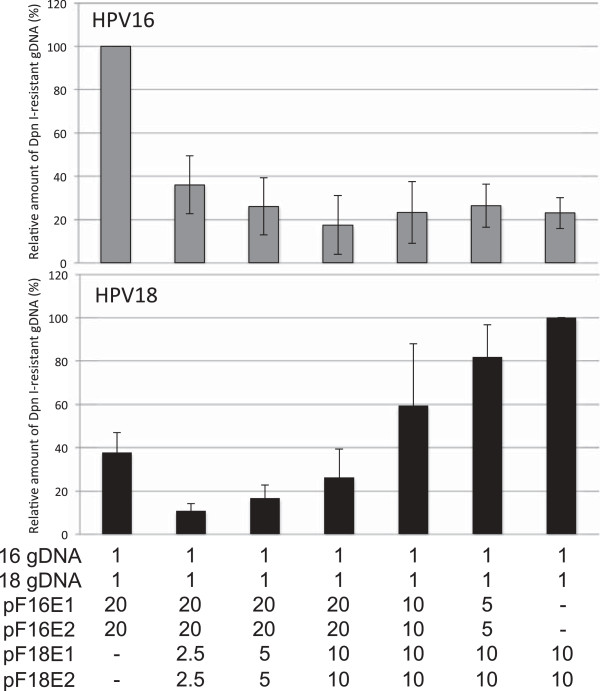
**Simultaneous replication of HPV16/18 genomes in the presence of E1/E2s of both types.** C33A cells were transfected with a mixture of HPV16 and HPV18 gDNAs (1 ng each) together with the indicated amounts (ng) of the expression plasmids. Three days after transfection, low molecular weight DNA was isolated by the Hirt procedure and digested with *Dpn*I. The *Dpn*I-resistant HPV16 (upper panel) and HPV18 (lower panel) gDNAs were quantified by real-time PCR and normalized to the luciferase gene. The level of the replication was presented as the relative amount of the *Dpn*I-resistant DNA compared to that obtained by the replication with the homologous E1/E2 alone. Each bar represents the average of three independent experiments with the standard deviation.

Replication of HPV16 and HPV18 genomes was suppressed by co-expression of the heterologous E1/E2 (Figure 3). C33A cells were transfected with a mixture of gDNAs and E1/E2 expression plasmids for both types to mimic simultaneous replication of HPV16 and HPV18 genomes in a co-infected cell. When cells were transfected with 20 ng of each pF16E1 and pF16E2 together with 10 ng of each pF18E1 and pF18E2, the replication levels of both types were reduced to around 20%. The inhibitory effect of HPV18 E1/E2 on HPV16 replication was more pronounced than that of HPV16 E1/E2 on HPV18 replication.

### Effects of heterologous E1 or E2 on HPV16/18 replication

**Figure 4:**
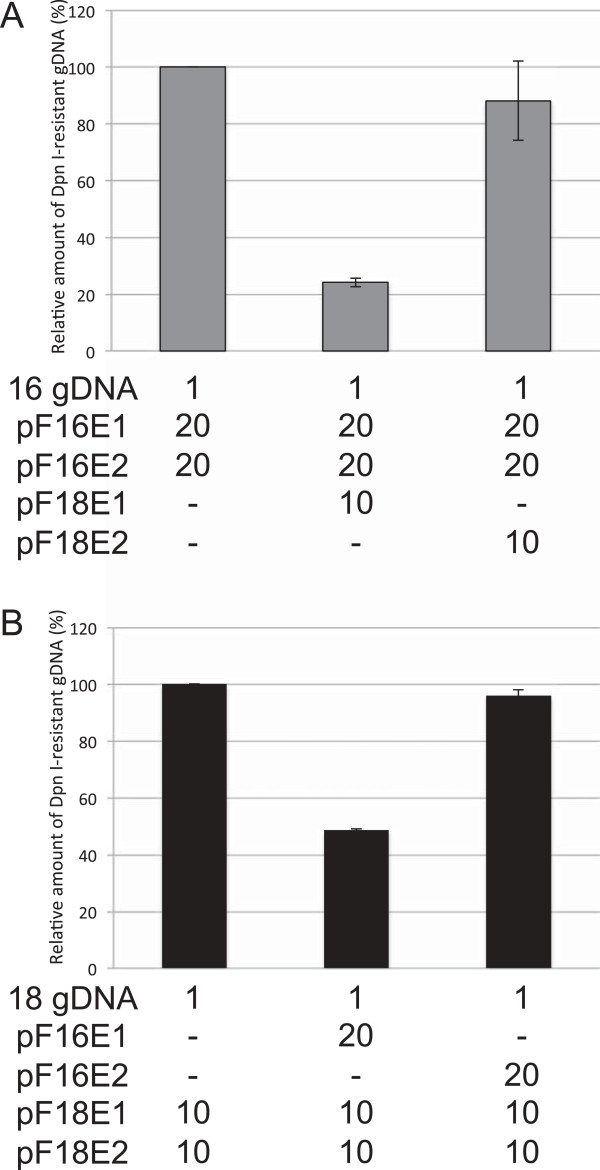
**Effects of heterologous E1 or E2 on HPV16/18 replication.** C33A cells were transfected with 1 ng of the HPV16 **(A)** or HPV18 **(B)** gDNAs together with the indicated amounts (ng) of the expression plasmids for E1/E2s. Three days after transfection, low molecular weight DNA was isolated by the Hirt procedure and digested with *Dpn*I. The *Dpn*I-resistant HPV gDNA was quantified by real-time PCR and normalized to that of the luciferase gene. The level of the replication was presented as the relative amount of the *Dpn*I-resistant DNA compared to that obtained by the replication with the homologous E1/E2 alone. Each bar represents the average of two independent experiments with the standard error of mean.

The heterologous E1, but not E2 inhibited HPV16 and HPV18 replication (Figure 4). HPV18 E1 inhibited HPV16 replication to the level observed in the presence of E1/E2s of both types (Figure 4A). The same was true for HPV16 E1 in HPV18 replication inhibition (Figure 4B). These results clearly indicate that the replication interference between HPV16 and HPV18 is mediated by the heterologous E1, not by E2.

### Effects of HPV16 E1 mutants on HPV18 replication

**Figure 5:**
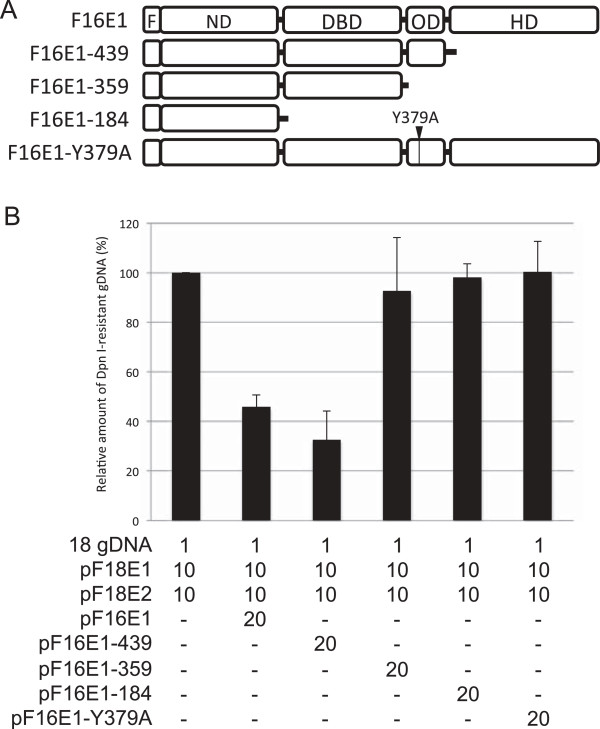
**Effects of HPV16 E1 mutants on HPV18 replication. (A)** Schematic representation of HPV16 E1 mutants. F, FLAG-tag; ND, N-terminal domain; DBD, DNA-binding domain; OD, oligomerization domain; HD, helicase domain. **(B)** C33A cells were transfected with 1 ng of the HPV18 gDNA together with the indicated amounts (ng) of the expression plasmids. Three days after transfection, low molecular weight DNA was isolated by the Hirt procedure and digested with *Dpn*I. The *Dpn*I-resistant HPV18 gDNA was quantified by real-time PCR and normalized to the luciferase gene. The level of the replication was presented as the relative amount of the *Dpn*I-resistant DNA compared to that obtained by the replication with the HPV18 E1/E2 alone. Each bar represents the average of two independent experiments with the standard error of mean.

To examine which functional domain of HPV16 E1 is responsible for the inhibition of HPV18 replication, we constructed expression plasmids for deletion mutants of HPV16 E1 (Figure 5A), and tested their capability to inhibit HPV18 replication.

As shown in Figure 5B, the OD of HPV16 E1 was essential for the inhibition of HPV18 replication, whereas the HD is dispensable. A deletion mutant F16E1-439, which lacks the HD, inhibited HPV18 replication as efficiently as full-length F16E1. In contrast, deletion mutants F16E1-359 and F16E1-184 failed to inhibit HPV18 replication. As expected, all of the deletion mutants completely lost the ability to support HPV16 replication (data not shown). Furthermore, an amino-acid substitution mutant F16E1-Y379A, which is supposed to negate the ability of E1 to oligomerize on single-stranded DNA and to bind to the replication origin [9,29], did not inhibit HPV18 replication. These results indicate that the OD of HPV16 E1 is responsible for the observed reduction in HPV18 replication efficiency.

### Physical interaction between E1s of HPV16/18 in C33A cells

**Figure 6:**
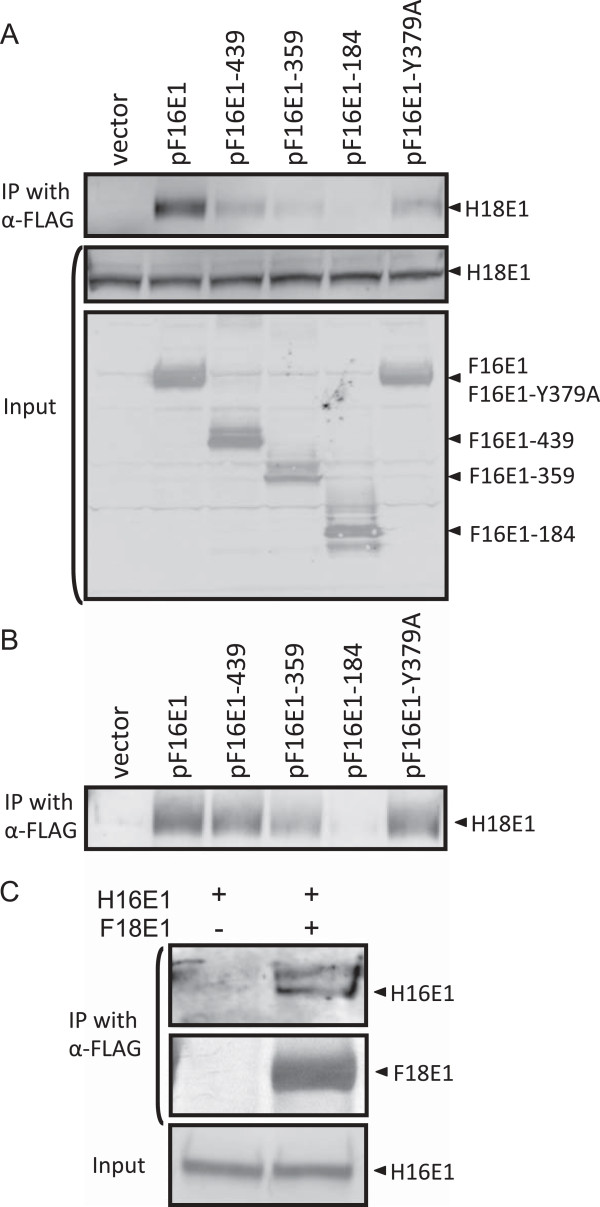
**Interaction between E1s of HPV16/18. (A)** C33A cells were transfected with the HPV18 gDNA together with the expression plasmids for hexahistidine (6×His)-tagged HPV18 E1 (pH18E1), the untagged HPV18 E2 (p18E2), and one of the FLAG-tagged HPV16 E1 mutants. Two days after transfection, the FLAG-tagged HPV16 E1s in cell lysates were immunoprecipitated with anti-FLAG antibody, and HPV18 E1 co-precipitated with HPV16 E1s was detected by Western blotting with anti-6×His antibody (upper panel). HPV18 E1 (middle panel) and HPV16 E1s (lower panel) in the total cell lysates were detected by Western blotting with anti-6×His and anti-FLAG antibodies, respectively. **(B)** C33A cells were transfected with pH18E1 and pF16E1 without HPV18 gDNA and p18E2, followed by immunoprecipitation and Western blotting as described above. **(C)** Purified 6×His-tagged HPV16 E1 (H16E1) was incubated with or without purified FLAG-tagged HPV18 E1 (F18E1), followed by immunoprecipitation with anti-FLAG antibody. H16E1 and F18E1 in the immunoprecipitates were detected by Western blotting with anti-6×His (upper panel) and anti-FLAG antibodies (middle panel), respectively. H16E1 in the input mixture was visualized with anti-6×His antibody (lower panel).

The requirement of the OD of HPV16 E1 for the inhibition of HPV18 replication strongly suggests that a physical interaction between the heterologous E1s is involved in the interference. Thus, we examined the interaction between E1s of HPV16 and HPV18 using co-immunoprecipitation assays. C33A cells were transfected with HPV18 gDNA together with expression plasmids for hexahistidine (6×His)-tagged HPV18 E1 (pH18E1), untagged HPV18 E2 (p18E2), and each of the HPV16 E1 mutants described above. Two days after transfection, F16E1 in cell lysates was immunoprecipitated with anti-FLAG antibody, followed by detection of H18E1 co-precipitated with F16E1 by Western blotting with anti-6×His antibody. H18E1 efficiently co-precipitated with F16E1 in the cell lysate (Figure 6A). Similarly H18E1 co-precipitated with F16E1 in the absence of HPV18 E2 and gDNA (Figure 6B), suggesting that the interaction is direct and not mediated by either HPV18 E2 or gDNA. H18E1 bound less efficiently to F16E1-439 and F16E1-359, but not to F16E1-184 (Figure 6A). Importantly, the OD mutant, F16E1-Y379A, showed reduced binding efficiency compared to F16E1.

The direct interaction between E1s of HPV16 and HPV18 was further confirmed by using purified E1 proteins: 6xHis-tagged HPV16 E1 (H16E1) and FLAG-tagged HPV18 E1 (F18E1). H16E1 was incubated with F18E1, and F18E1 in the mixture was immunoprecipitated with anti-FLAG antibody, followed by Western blotting with anti-6×His antibody. Clearly H16E1 co-precipitated with F18E1 (Figure 6C), indicating that HPV16 E1 directly binds to HPV18 E1.

Taken together, these results strongly suggest that HPV16 E1 interacts with HPV18 E1 through the region containing the DBD, OD, and HD, and that the OD is an important determinant for efficient interaction.

## Discussion

In this study using cell-based transient replication assays we found replication interference between HPV16 and HPV18 and explored the underlying molecular mechanisms. We examined replication of HPV16/18 genomes under conditions of exogenous expression of E1/E2 for both types from the Cytomegalovirus promoter because expression of E1/E2 from native HPV promoters is too weak to support HPV genome replication in C33A cells. Although these experimental conditions do not necessarily reflect a natural context of HPV transcription, the results obtained in this study provide a novel mechanistic insight into inter-type HPV replication interference, which could not be addressed in the previous study using transfection of HPV genomes alone [20].

The mutational analysis of HPV16 E1 indicates that the OD, which is responsible for the E1-E1 interaction [9,10], is essential for the inhibition of HPV18 replication. In particular, the inability of the OD mutant F16E1-Y379A to inhibit HPV18 replication strongly implies that the OD-mediated interaction between HPV16 and HPV18 E1s is critical for replication interference. On the other hand, the deletion mutant F16E1-439, which lacks the HD and shows reduced binding (at a similar level to the OD mutant), still retains the capability to inhibit HPV18 replication, suggesting that the binding between HPV16 and HPV18 E1s via the HD does not contribute to the replication interference. Furthermore, because the HD is essential for helicase activity, as well as E1 binding to E2 [30-32], the results clearly indicate that the enzymatic activity of E1 and its binding to E2 are not involved in replication inhibition. It is also worth noting that the replication interference is not simply due to competition between heterologous E1/E2s for origin binding because E1/E2s of HPV16 and HPV18 supported genome replication of heterologous types (Figure 2).

**Figure 7:**
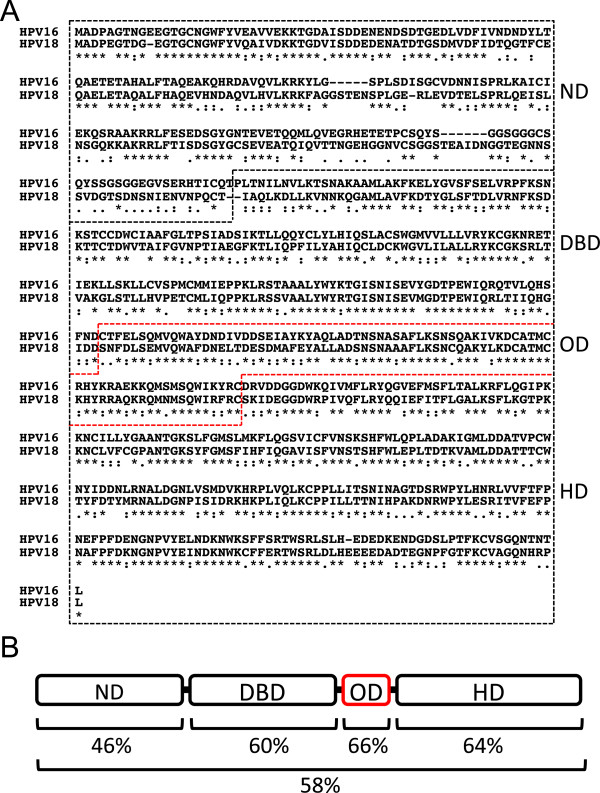
**Amino acid sequence similarity between HPV16/18 E1s. (A)** The amino acid sequence alignment between HPV16/18 E1s using MAFFT [33]. Identical residues are indicated with asterisks (*). Highly conserved residues are indicated with colons (:). Semi-conserved residues are indicated with periods (.). **(B)** The amino acid sequence identities of the ND (amino acids 1 to 190 of HPV16 E1 and 1 to 197 of HPV18 E1), DBD (amino acids 191 to 352 of HPV16 E1 and 198 to 359 of HPV18 E1), OD (amino acids 353 to 430 of HPV16 E1 and 360 to 437 of HPV18 E1), HD (amino acids 431 to 649 of HPV16 E1 and 438 to 657 of HPV18 E1), or the entire regions (amino acids 1 to 649 of HPV16 E1 and 1 to 657 of HPV18 E1) between HPV16/18 E1s are presented.

A comparison of amino acid sequences between HPV16 and HPV18 E1s reveals that the OD is the most conserved region between the two (Figure 7A). The amino acid identities in the ND, DBD, OD, and HD are 46, 60, 66, and 64% homologous, respectively (Figure 7B). The high sequence similarity observed between ODs strongly supports the possibility of heterooligomer formation between the two types. Collectively, these results implicate E1 heterooligomer formation via the OD as the molecular mechanism behind replication inhibition.

We propose a model in which HPV16 and HPV18 E1 heterooligomers do not have proper configurations and are deficient in the ability to support genome replication for both HPV16 and HPV18. Since the amino acid sequence in the ND is less conserved between HPV16 and HPV18 E1s (Figure 7), it may exert a negative effect on the replication activities of E1 heterooligomers, although the exact functions of the ND in the conformation of E1 homooligomers are not yet well understood. Alternatively, it is possible that the E1 heterooligomers are unable to bind efficiently to the replication origin or unwind double-stranded DNA, and/or that they are deficient in recruiting cellular factors required for HPV replication, such as DNA polymerase alpha [34], replication protein A [35], and topoisomerase I [36].

Serological studies have shown that seropositivities for HPV6 and HPV11 significantly antagonize the development of HPV16-related cervical cancer [37,38]. Interestingly, these studies also demonstrated a tendency for antagonistic interactions between HPV16 and HPV18. Seropositivity for HPV18 reduces the risk of HPV16-related cervical cancer, although statistical significance was not achieved. Although the underlying mechanism of such serological interaction is not clear, interference of genome replication between HPV16 and other HPV types may be involved, as observed in this study. It is unlikely that antibodies against HPV6, HPV11, or HPV18 capsids protect women from infection with HPV16, because antisera against these types do not cross-neutralize HPV16 in vitro [39]. It will thus be of interest to investigate whether HPV6 and HPV11 interfere with HPV16 replication in transient replication assays such as those described here.

Interference between multiple HPV types has been also proposed in several pathological studies. Genomes of different HPV types were detected in distinct, non-overlapping areas of the same genital tissue by in situ hybridization [40-42]. When cervical tumor biopsies, in which genomes of both HPV16 and HPV18 were detected by PCR, were analyzed by fluorescence in situ hybridization, the integrated form of either HPV16 or HPV18 was detected in an individual cell [43]. Although the genomes of HPV1 and HPV63 were detected in the same cell of a plantar wart, only a cytopathogenic effect typical of HPV63 infection was seen in the cell [44].

Our experimental results and previous observations reported by others indicate that interactions between different HPV types during their life cycle may affect the propagation, pathogenesis, and evolution of HPVs.

## Conclusions

This study indicates that co-infection of a single cell with HPV16 and HPV18 results in replication interference between them, and that interaction between the heterologous E1s is responsible for the interference.

## Materials and methods

### Plasmids and HPV genomes

The codon-optimized E1 and E2 genes of HPV16 and HPV18 were synthesized by Life Technologies (Carlsbad, CA, USA) and cloned into the *Not*I site of pCMV, which had been created by removing the β-galactosidase gene from pCMVβ (Clontech, Mountain View, CA, USA). The FLAG-tag sequence was added to the 5′-terminus of each gene in pCMV by using PCR to produce pF16E1, pF16E2, pF18E1, and pF18E2. The 6xHis-tag sequence was added to the 5′-terminus of the HPV18 E1 gene in pCMV to produce pH18E1. A stop codon was introduced into pF16E1 by using PCR to produce expression plasmids for HPV16 E1 deletion mutants; pF16E1-439, pF16E1-359, and pF16E1-184 express amino acid (aa) 2 to 439, aa 2 to 359, and aa 2 to 184 of the HPV16 E1, respectively. The codon 379 of the E1 gene in pF16E1 was changed from TAC to GCA by using PCR to produce pF16E1-Y379A, which expresses HPV16 E1 with amino acid substitution of tyrosine to alanine at aa residue 379.

The circularized HPV16 and HPV18 gDNAs were prepared by in vitro self-ligation of linear viral genomes. The full-length genomes of HPV16 and HPV18 were released from the cloned vector pUC18 by digestion with *Bam*HI and *Eco*RI, respectively. The linearized genomes were incubated with T4 DNA ligase at final concentration 2.5 μg/ml of DNA for overnight at 4°C. The ligated DNAs were purified and concentrated by using the QIAprep Spin Miniprep Kit (Qiagen GmbH, Hilden, Germany).

### Cell culture

C33A cervical carcinoma cells were cultured in Dulbecco’s modified minimal essential medium (DMEM) supplemented with 10% fetal bovine serum (FBS) and grown in 5% CO_2_ at 37°C.

### Western blot

C33A cells (1 × 10^6^ cells) were grown in a 6-well plate for 6 h and then transfected with the expression plasmids for FLAG-tagged E1/E2 of HPV16/18 using the FuGene HD (Roche Diagnostics, Indianapolis, IN, USA). Two days after transfection the cells were lysed in SDS-sample buffer, followed by boiling of the cell lysate for 5 min. The extracted proteins were separated by SDS-PAGE and transferred to a PVDF membrane (Life Technologies). After blocking the membrane with phosphate-buffered saline (PBS) containing 0.02% Tween-20 and 5% skimmed milk, FLAG-tagged E1/E2s were probed with rabbit polyclonal anti-FLAG antibody (Sigma-Aldrich Co. St. Louis, Mo, USA) and horseradish peroxidase conjugated anti-rabbit IgG goat antibody (Santa Cruz Biotechnology, Inc., Santa Cruz, CA, USA). The immunoreactive proteins were visualized using the Pierce Western Blotting Substrate Plus (Thermo Fisher Scientific Inc. Waltham, MA, USA) and the Typhoon 9410 Image Scanner (GE Healthcare, Piscataway, NJ, USA).

### Transient replication assay

C33A cells (2 × 10^5^ cells) were grown in a 24-well plate for 6 h and then transfected with HPV gDNAs and the expression plasmids for E1/E2 together with 1 ng of pGL3-Basic (Promega, Madison, WI, USA), which lacks a replication origin for HPV, using the FuGene HD. The total amount of transfected DNA was adjusted to 400 ng with the empty vector, pCMV. The next day of the transfection, the medium was changed to DMEM containing 10% FBS. Three days after transfection low molecular weight DNA was isolated by the Hirt procedure. Briefly, 200 μl of lysis solution (10 mM Tris–HCl [pH 8.0], 10 mM EDTA [pH 8.0], and 1% SDS) was added to the cells, followed by cell lysis for 10 min at room temperature with gentle agitation. Fifty μl of 5 M NaCl was then added to the cell lysate, and incubated overnight at 4°C. The mixture was centrifuged at 50,000 × g for 30 min at 4°C to precipitate proteins and genomic DNA of the cells. The HPV gDNAs were isolated from the supernatant using the QIAamp DNA Blood Mini Kit (Qiagen GmbH), and eluted with 50 μl of elution buffer (10 mM Tris–HCl [pH 8.0] and 1 mM EDTA). To digest the transfected DNA, 2 μl of the DNA sample was incubated with 10 U of *Dpn*I in 20 μl of 1 × reaction buffer (New England Biolabs, Ipswich, MA, USA) for 2 h at 37°C, followed by heat inactivation of *Dpn*I at 80°C for 20 min. The amounts of *Dpn*I-resistant HPV16/18 gDNAs were quantified by real-time PCR with HPV16/18 type-specific primers and SYBR-green dye. Total 20 μl of a PCR reaction mixture containing 2 μl of the *Dpn*I-digested sample, 10 μl of SYBR Green Realtime PCR Master Mix (TOYOBO CO., LTD, Osaka, Japan), and 0.4 μM of each primer was subjected to real-time PCR analysis using the LightCycler 480 (Roche Diagnostics). The nucleotide sequences of primers are as follows: forward primer for HPV16, 5′-CACCTCCAGCACCTAAAGAA; reverse primer for HPV16, 5′-TTGCGTCCTAAAGGAAACTG; forward primer for HPV18, 5′-GATGTGAGAAACACACCACA; reverse primer for HPV18, 5′-GCAGTGAAGTGTTCAGTTCC; forward primer for the luciferase gene, 5′-AGGCGAACTGTGTGTGAGAG; reverse primer for the luciferase gene, TTCAGGCGGTCAACGATGAA. Each amplicon of the HPV16/18 genomes contains two *Dpn*I sites, whereas that of the luciferase gene has no *Dpn*I site. The amounts of *Dpn*I-resistant HPV gDNA were normalized to those of the luciferase gene.

### Immunoprecipitation

C33A cells (1 × 10^6^ cells) were grown in a 6-well plate for 6 h and then transfected with HPV18 gDNA, pH18E1, and p18E2, together with one of the expression plasmids for HPV16 E1 mutants, using the FuGene HD. Two days after transfection the cells were lysed in 100 μl of RIPA buffer (25 mM Tris–HCl [pH7.6], 150 mM NaCl, 1% NP-40, 1% sodium deoxycholate, and 0.1% SDS), followed by centrifugation of the cell lysate at 10,000 × g for 10 min at 4°C. The supernatant was diluted 4-fold with PBS, and precleared with 20 μl of Dynabeads protein G magnetic beads (Life Technologies) for 30 min at 4°C. The precleared lysate was further incubated with 2 μg of the rabbit polyclonal anti-FLAG antibody (Sigma-Aldrich Co.) and 20 μl of the protein G magnetic beads for 2 h at 4°C. The beads were then washed with PBS containing 0.02% Tween-20 four times, and the bound proteins were eluted by boiling the beads in 20 μl of SDS-sample buffer. The recovered proteins were resolved by SDS-PAGE and transferred to a PVDF membrane. 6×His-tagged HPV18 E1 on the membrane was probed by mouse monoclonal anti-6×His antibody (Qiagen GmbH) and detected with horseradish peroxidase conjugated anti-mouse IgG goat antibody (Santa Cruz Biotechnology, Inc.) as described above.

6×His-tagged HPV16 E1 (H16E1) protein was prepared from bacterial cells. Briefly, the H16E1 gene was cloned into pET-30 (Novagen, Madison, WI, USA), and the H16E1 protein was purified from *Escherichia coli* BL21(DE3) pLysS that had been transformed with the expression plasmid as described previously [45]. FLAG-tagged HPV18 E1 (F18E1) protein was prepared from insect cells by using the baculovirus expression system (Life Technologies). Briefly, the F18E1 gene was cloned into pFastBac1 (Life Technologies), and the resultant plasmid was introduced into *E. coli* DH10Bac-competent cells (Life Technologies) to obtain recombinant Bacmid DNA. A recombinant baculovirus expressing F18E1 was produced in Sf9 cells transfected with the recombinant Bacmid DNA. The F18E1 protein was purified from Sf9 cells that had been infected with the recombinant baculovirus as described previously [45]. For binding reaction, 1 μg of H16E1 and 4 μg of F18E1 were diluted with 200 μl of binding buffer (20 mM Tris–HCl [pH8.0], 150 mM NaCl, 2 mM MgCl2, and 0.01% NP-40), and incubated with 4 μl of the rabbit polyclonal anti-FLAG antibody (Sigma-Aldrich Co.) and 15 μl of the protein G magnetic beads (Life Technologies) for 1 h at 4°C. The beads were then washed with the binding buffer four times, and the bound proteins were analyzed by Western blotting as described above.

## Abbreviations

HPV: Human papillomavirus; E1BS: E1 binding site; E2BS: E2 binding site; ND: N-terminal domain; DBD: DNA-binding domain; OD: Oligomerization domain; HD: Helicase domain; aa: Amino acid; DMEM: Dulbecco’s modified minimal essential medium; FBS: Fetal bovine serum; PBS: Phosphate-buffered saline.

## Competing interests

The authors declare that they have no competing interests.

## Authors’ contributions

SM and IK designed this study and drafted the manuscript. SM performed experiments. RKM and IK technically supported the experiments. SM, RKM, YI, TT, and IK revised the manuscript critically. All authors have read and approved the final manuscript.
